# Radiotherapy and chemoradiotherapy for postoperative recurrence in patients with esophageal squamous cell carcinoma

**DOI:** 10.1002/cam4.70108

**Published:** 2024-08-19

**Authors:** Qing Liu, Xue‐Hua Tu, Rui‐Xuan Yu, Hong‐Ying Wen, Xiao‐Guang Guo, Dai‐Yuan Ma, Kai‐Yuan Jiang, Dong Tian

**Affiliations:** ^1^ Integrated Care Management Center West China Hospital, Sichuan University Chengdu China; ^2^ Anesthesia Operation Center of West China Hospital/West China School of Nursing, Sichuan University Chengdu China; ^3^ Department of Thoracic Oncology, Cancer Center West China Hospital, Sichuan University Chengdu China; ^4^ Department of Cardiothoracic Intensive Care Unit Affiliated Hospital of North Sichuan Medical College Nanchong China; ^5^ Department of Pathology Nanchong Central Hospital Nanchong China; ^6^ Department of Oncology Affiliated Hospital of North Sichuan Medical College Nanchong China; ^7^ Department of Surgery Tohoku University Graduate School of Medicine Sendai Japan; ^8^ Department of Thoracic Surgery West China Hospital, Sichuan University Chengdu China

**Keywords:** chemoradiotherapy, esophageal squamous cell carcinoma, prognosis, radiotherapy, recurrence, toxicity

## Abstract

**Background:**

The optimal treatment for esophageal squamous cell carcinoma (ESCC) patients with postoperative recurrence remains controversial. We aimed to evaluate the effects of radiotherapy (RT) and chemoradiotherapy (CRT) on postoperative recurrence in ESCC patients.

**Methods:**

Recurrence ESCC patients who received salvage RT and CRT from January 2015 to January 2019 were retrospectively reviewed. Post‐recurrence survival (PRS) and prognostic factors were evaluated by Kaplan–Meier and Cox proportional hazards models, respectively. Subgroup analyses were performed based on pathological lymph node (LN) status (negative/positive) to evaluate the differences in salvage treatments and toxic reaction.

**Results:**

A total of 170 patients were enrolled, with a median age of 60 years (range 43–77). No significant difference was found in the median PRS between the salvage RT and CRT groups (*p* > 0.05). Multivariate analysis revealed that TNM stage III and IV, macroscopic medullary type, and distant metastasis recurrence pattern were independent prognostic factors (all *p* < 0.05) for PRS. Salvage treatment was not associated with PRS (*p* = 0.897). However, in patients with negative LN, CRT was associated with prolonged survival (*p* = 0.043) and had no significant differences in toxic reactions compared to RT (*p* = 0.924). In addition, RT showed better prognoses (*p* = 0.020) and lower toxic reactions (*p* = 0.030) than CRT in patients with positive LNs.

**Conclusions:**

Based on prognosis and toxic reactions, ESCC recurrence patients with negative LNs could benefit from CRT, but RT should be recommended for patients with positive LNs.

## 1 INTRODUCTION

Esophageal cancer (EC) ranks as the seventh most common malignancy in humans and the sixth leading cause of cancer‐related mortality globally.^1^, ^2^ Histologically, EC is mainly defined as esophageal squamous cell carcinoma (ESCC) and esophageal adenocarcinoma. The incidence and histology of each vary by geographical location, and ESCC remains the predominant histological subtype in Asian countries.^3^ Although radical esophagectomy with lymph node (LN) dissection is the primary treatment for ESCC, the therapeutic outcomes are not satisfactory, with 5‐year post‐recurrence survival (PRS) rates ranging from 15%–47%.^4^, ^5^ The poor long‐term prognosis has been attributed to postoperative recurrence and metastasis, with a recurrence rate of 23%–53%.^6^, ^7^, ^8^

Regarding the recurrence of ESCC, 22%–68% of patients develop LN recurrence/anastomotic recurrence, and 12%–51% present distant metastasis.^9^ Several treatment modalities, including chemotherapy (CT), radiotherapy (RT), chemoradiotherapy (CRT), and surgery, are available for recurrent ESCC.^10^, ^11^ CT alone has been demonstrated to have a significantly worse effect than other treatments.^12^, ^13^ According to previous reports, salvage RT or esophagectomy are recommended for patients with anastomotic recurrence.^14^, ^15^ Regarding LN recurrence and distant organ metastasis, salvage RT and CRT are considered acceptable options.^16^, ^17^ Nevertheless, previous studies have reported that the 5‐year PRS with CRT was 4.3%, with median PRS times of 16 months and 22 months for patients who received RT and CRT, respectively.^2^, ^18^ The optimal therapeutic strategy for recurrent ESCC, especially LN recurrence and distant metastasis, has not been confirmed in the National Comprehensive Cancer Network (NCCN) guidelines.^2^, ^8^ LN status (positive/negative) is the most important prognostic factor for patients with ESCC. Additional adjuvant therapy is usually performed in patients with pathologically confirmed positive LNs, but the optimal treatment for patients with postoperative recurrence and different LN statuses is still unknown.^19^, ^20^ To enable individualized treatment, clarifying the prognostic factors affecting PRS and determining the effectiveness of remedies for recurrence are highly important.

In this study, we sought to explore the prognostic factors for PRS and evaluate the effect of RT and CRT as salvage treatments in patients with recurrent ESCC after radical esophagectomy. Moreover, we further performed subgroup analysis based on postoperative LN status to identify differences in efficacy and toxicity between RT and CRT.

## 2 METHODS

### 2.1 Study populations

ESCC patients who developed postoperative recurrence and subsequently underwent RT or CRT in the Department of Oncology, Affiliated Hospital of North Sichuan Medical College from January 2015 to January 2019 were retrospectively reviewed. The inclusion criteria were as follows: (1) pathologically confirmed ESCC; (2) recurrence after esophagectomy (R0 resection) with LN dissection; (3) postoperative recurrence confirmed by enhanced computed tomography; if necessary, positron emission tomography and pathological examination of biopsy were performed; and (4) received salvage treatments (RT/CRT). The study was performed in accordance with the Declaration of Helsinki and was authorized by the Ethics Committees and Review Board of the Affiliated Hospital of North Sichuan Medical College (No. 2020ER181‐1). The requirement for patient consent was removed due to the retrospective nature of the present study.

### 2.2 Data collection and definition

Patient data including sex (male/female), age (≤60/>60), tumor location (upper/middle/lower), TNM stage (I/II/III/IV), surgical approach (left/right), macroscopic type (ulcerative/fungating/constrictive/medullary), recurrence pattern (LN recurrence/distant metastasis), disease‐free survival (DFS) (≤1 year/>1 year), salvage treatment (RT/CRT), toxicity (grade 1 and 2/grade 3 and 4), PRS and other clinical characteristics for recurrent ESCC were investigated. The pathological results were re‐evaluated by a pathologist (XGG) according to the 8th edition American Joint Committee on Cancer (AJCC) and the Union for International Cancer Control (UICC) guidelines.

The recurrence patterns in this study included LN recurrence and distant metastasis (metastasis to solid organs or recurrence in the pleura or peritoneal cavity). DFS was defined as the period from the first day after surgery to a recurrence. PRS was defined as the interval between initial recurrence and either death from any cause, loss to follow‐up, or last follow‐up.

Treatment toxicities were assessed by reviewing medical reports and laboratory results according to Radiation Therapy Oncology Group (RTOG) guidelines.^21^ Adverse events were graded based on the National Cancer Institute Common Toxicity Criteria Adverse Events Version 5.0 (CTCAE V5.0).^22^ Patients were divided into grade 1/2 and grade 3/4 subgroups according to the toxicity criteria above.

### 2.3 Treatment

Forty cases received neoadjuvant CT with the regimen of 5‐fluorouracil (800 mg/m^2^ infusions on Days 1–5) and cisplatin (80 mg/m^2^ on Day 1) for 2–3 cycles. Radical esophagectomy via left (Sweet esophagectomy) or right (McKeown or Ivor‐Lewis esophagectomy) approaches were conducted with at least a two‐field (thoracic and abdominal) lymphadenectomy. Patients with suspected LN involvement in the cervical field assessed by preoperative computer tomography and ultrasound underwent three‐field LN dissection. After surgery, 114 patients received CT with paclitaxel (150 mg/m^2^, intravenously on Day 1) and cisplatin (50 mg/m^2^ on Day 1) every 2 weeks. Ten patients underwent RT with a total radiation dose of 50–54 Gy administered in fractions of 1.8–2.0 Gy, 5 d/wk. Forty‐six patients did not receive any adjuvant treatment.

All patients with confirmed ESCC recurrence received salvage RT or CRT. According to the Chinese Guidelines for the Treatment of Esophageal Cancer, patients with local recurrence could receive either RT or CRT, while CRT is preferred for those with distant metastases, with RT as a secondary option due to considerations of tolerance and toxicity for individuals. The treatment plan is also adjusted based on the individual status. Sixty‐two patients underwent RT alone. One hundred and eight patients received CT consisting of 5‐fluorouracil (700 mg/m^2^ infusions on Days 1–5) and cisplatin (20 mg/m^2^ on Days 1–5 or 40 mg/m^2^ on Days 1–3), and RT. A total radiation dose of 50–60 Gy to gross tumor volume and LN recurrence area (bilateral supraclavicular region/ mediastinum region/abdominal region) was administered in fractions of 1.8–2.0 Gy/d, 5 d/wk. Additional irradiation was primarily given to address severe pain arising from bone metastasis or to treat severe symptoms in other metastatic organs. For the 10 patients who received adjuvant RT postoperatively, the interval between treatments was more than 1 year, and the dose was controlled within the range of 30–40 Gy.

### 2.4 Follow‐up

Patients were evaluated at the end of the first month, at 3‐month intervals for the first year, at 6‐month intervals for the second year and third year, and annually thereafter. Examination included barium swallow, chest CT, and neck and abdominal ultrasound. If recurrence was suspected, enhanced CT was performed, and PET/CT or pathological examination was conducted if necessary.

### 2.5 Statistical analysis

All statistical analyses were performed using IBM SPSS Statistics (version 25.0 Inc., Chicago, IL, USA) and the R programming language (version 4.0.2, Vienna, Austria).

The continuous and categorical variables are summarized using descriptive statistics. The PRS was estimated using the Kaplan–Meier method with the log‐rank test. Cox proportional hazards models were administered to identify independent prognostic factors for PRS. A binary logistic regression model was used to determine the risk factors for toxicity reaction. Only variables in the univariate analysis with a *p* < 0.050 were included in the multivariate analysis. Subgroup analyses of postoperative LN status were further performed to determine differences in prognosis and toxicity between salvage treatment methods. The hazard ratio (HR), odds ratio (OR), and 95% confidence interval (CI) were calculated. Differences were considered statistically significant when *p* < 0.050.

## 3 RESULTS

### 3.1 Patients' characteristics and recurrence information

**FIGURE 1:**
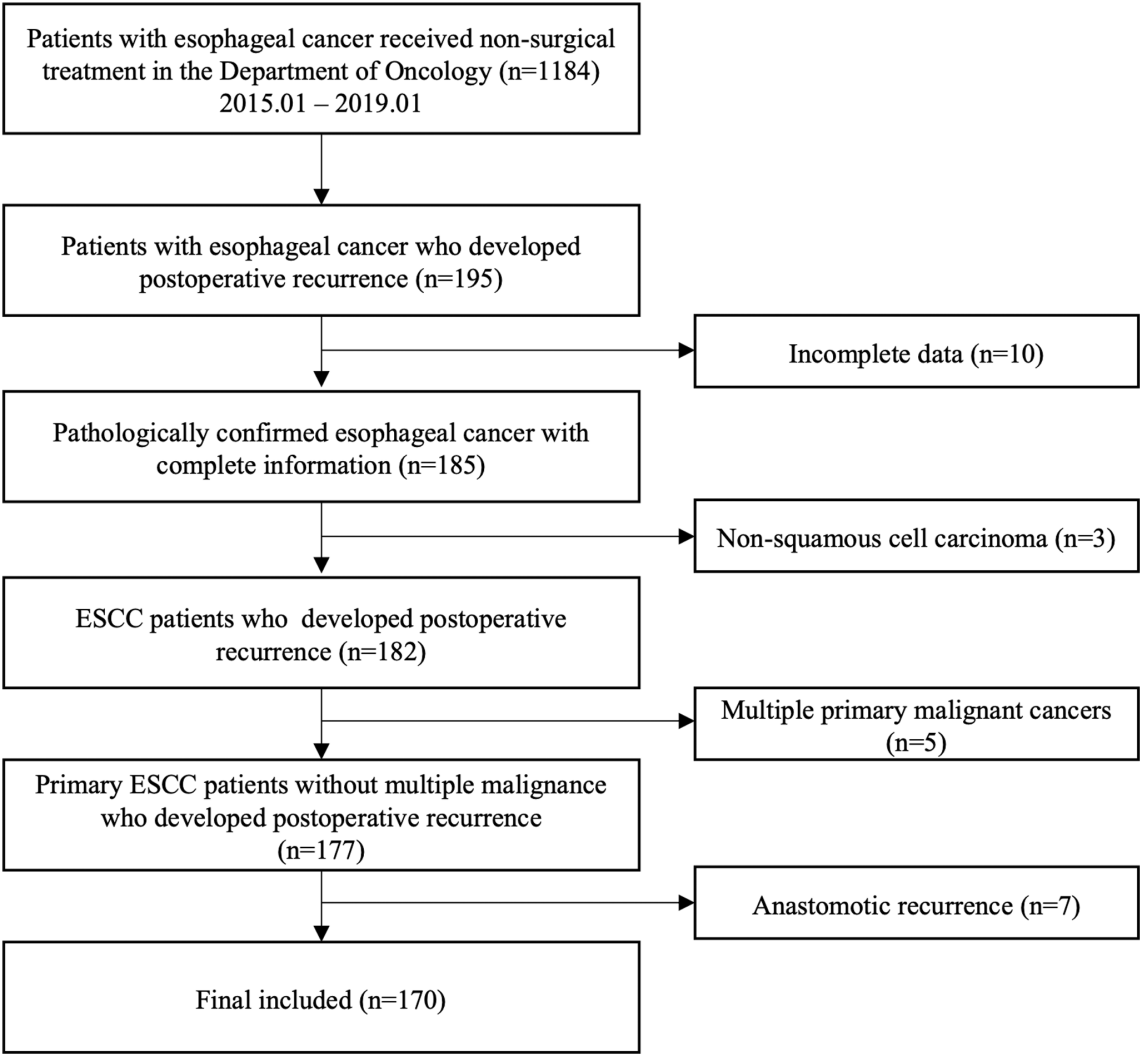
The process of identification for the retrospective study. ESCC, esophageal squamous cell cancer; LN, lymph node.

Initially, there were 1184 consecutive patients with EC received treatment at Department of Oncology. Then, 195 patients with recurrent EC were enrolled. We excluded patients with other or multiple primary malignant cancers, anastomotic recurrence, and incomplete data. Finally, a total of 170 (92 males and 78 females), with a median age of 60 years (range, 43–77) were included in this study (Figure 1). Of these patients, 62 received salvage RT alone (36.5%), and 108 underwent salvage CRT (63.5%). Neoadjuvant therapy was administered to 45 patients (26.5%), and most patients (124, 72.9%) received adjuvant therapy after surgery. Esophagectomy with right approach was performed in 91 patients (53.5%), and the remaining 79 patients (46.5%) underwent left thoracic approach. The ulcerative type of ESCC was observed in nearly half of the patients (77, 45.3%), followed by the fungating type (46, 27.1%), the medullary type (31, 18.2%), and the constrictive type (16, 9.4%). Of the included patients, 80 (47.1%) developed recurrence within 1 year. LN recurrence (111 cases, 65.3%) was the main recurrence pattern, followed by distant metastasis (59 cases, 34.7%). There were 42 cases of cervical LN recurrence, 63 cases of mediastinal LN recurrence, and 20 cases of abdominal LN recurrence, with 98 cases (88.3%) of single‐site recurrence and 13 cases (11.7%) of multiple‐site recurrence. In the distant metastasis group, there were 31, 26, 14, 5, 4, 4, and 1 cases of distant metastases to the lung, bone, liver, brain, spleen, kidney, and testis respectively, with 39 cases (66.1%) of single metastases and 20 cases (33.9%) of multiple metastases. Based on LN status after radical esophagectomy with lymphadenectomy, the patients were assigned to either a negative LNs (*n* = 88) or positive LNs (*n* = 82) subgroup. More detailed clinicopathologic information is shown in Table 1.

**TABLE 1:** Clinicopathologic characteristics.

Variables	No. of patients (percentage)		
	All patients (*n* = 170)	LN− Subgroup (*n* = 88)	LN+ Subgroup (*n* = 82)
Age (year)			
≤60	88 (51.8%)	42 (47.7%)	46 (56.1%)
>60	82 (48.2%)	46 (52.3%)	36 (43.9%)
Sex			
Male	92 (54.1%)	45 (51.1%)	47 (57.3%)
Female	78 (45.9%)	43 (48.9%)	35 (42.7%)
Neoadjuvant chemotherapy			
Yes	45 (26.5%)	27 (30.7%)	18 (22.0%)
No	125 (73.5%)	61 (69.3%)	64 (78.0%)
Adjuvant therapy			
Yes	124 (72.9%)	48 (54.5%)	76 (92.7%)
No	46 (27.1%)	40 (45.5%)	6 (7.3%)
Surgery approach			
Left	79 (46.5%)	40 (45.5%)	39 (47.6%)
Right	91 (53.5%)	48 (54.5%)	43 (52.4%)
Tumor location			
Upper	28 (16.5%)	16 (18.2%)	12 (14.6%)
Middle	110 (64.7%)	58 (65.9%)	52 (63.4%)
Lower	32 (18.8%)	14 (15.9%)	18 (22.0%)
Lymph node metastasis			
Present	82 (48.2%)	0 (0.0%)	82 (100.0%)
Absent	88 (51.8%)	88 (100.0%)	0 (0.0%)
TNM stage			
I	24 (14.1%)	24 (27.3%)	0 (0.0%)
II	56 (32.9%)	54 (61.4%)	2 (2.4%)
III	74 (43.5%)	7 (7.9%)	67 (81.7%)
IV	16 (9.5%)	3 (3.4%)	13 (15.9%)
Macroscopic types			
Ulcerative type	77 (45.3%)	39 (44.3%)	38 (46.3%)
Fungating type	46 (27.1%)	22 (25.0%)	24 (29.3%)
Constrictive type	16 (9.4%)	11 (12.5%)	5 (6.1%)
Medullary type	31 (18.2%)	16 (18.2%)	15 (18.3%)
Disease‐free survival			
≤1 year	80 (47.1%)	30 (34.1%)	50 (61.0%)
>1 year	90 (52.9%)	58 (65.9%)	32 (39.0%)
Patterns of recurrence			
Lymph node recurrence	111 (65.3%)	65 (73.9%)	46 (56.0%)
Distant metastasis with LNM	59 (34.7%)	23 (26.1%)	36 (44.0%)
Salvage treatment			
RT	62 (36.5%)	29 (33.0%)	33 (40.2%)
CRT	108 (63.5%)	59 (67.0%)	49 (59.8%)
Toxicity			
Grade 1/2	92 (54.1%)	54 (61.4%)	38 (46.3%)
Grade 3/4	78 (45.9%)	34 (38.6%)	44 (53.7%)

Abbreviations: CRT, chemoradiotherapy; ESCC, esophageal squamous cell carcinoma; LN−, negative lymph node status; LN+, positive lymph node status; LNM, lymph node metastasis; RT, radiotherapy.

### 3.2 Survival analysis

**FIGURE 2:**
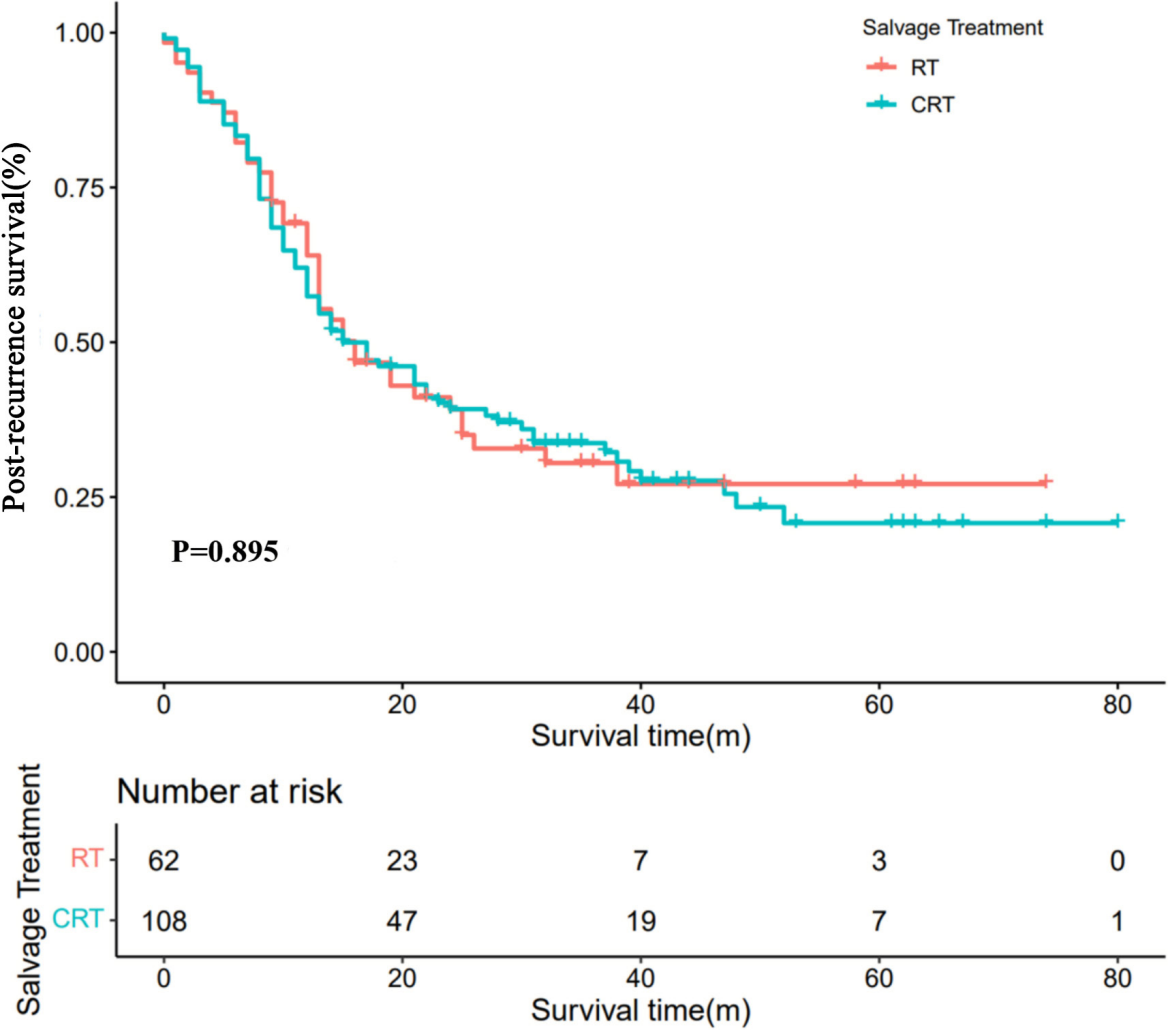
Kaplan–Meier curves of salvage treatment on PRS outcomes in ESCC. RT, radiotherapy; CRT, chemoradiotherapy; PRS, post‐recurrence survival.

The median time to recurrence was 15.5 months (range: 1–124 months). The median follow‐up duration was 43 months (range: 0–80 months). The 1‐, 3‐, and 5‐year PRS rates of the RT and CRT groups were 64% versus 57%, 27% versus 32%, and 3.3% versus 5.6%. No significant differences in median PRS (16 months vs. 15 months, *p* = 0.897, Figure 2) were observed between patients who received RT and CRT.

No significant differences were observed in the survival curve between patients with single and multiple sites LN recurrences (*p* = 0.930, Figure S1A) or in distant metastasis at single or multiple sites (*p* = 0.140, Figure S1B).

### 3.3 Prognostic factors

In the univariate analysis, TNM stage, macroscopic type, DFS (≤1 year vs. >1 year), and recurrence pattern were associated with PRS (*p* < 0.050). Sex, age, adjuvant therapy, tumor location, and salvage treatment were not related to PRS (*p* ≥ 0.050). The multivariate analysis indicated that advanced TNM stage III and IV (HR = 2.133, 95% CI = 1.070–4.251, *p* = 0.031; HR = 2.316, 95% CI = 1.010–5.310, *p* = 0.047), macroscopic medullary type (HR = 2.167, 95% CI = 1.298–3.620, *p* = 0.003), and distant metastasis recurrence pattern (HR = 1.682, 95% CI = 1.138–2.485, *p* = 0.009) were independent adverse prognostic factors (Table 2). The Kaplan–Meier curves of TNM stage, macroscopic types, DFS, and recurrence pattern are shown in Figure S2.

**TABLE 2:** Cox regression analysis for ESCC patients with recurrence.

Parameters	Univariate		Multivariate	
	HR (95% CI)	*p*‐value	HR (95% CI)	*p*‐value
Sex (male/female)	0.745 (0.518–1.073)	0.114		
Age (≤60/>60)	1.008 (0.985–1.031)	0.504		
Neoadjuvant chemotherapy (yes/no)	1.137 (0.761–1.700)	0.531		
Adjuvant therapy (yes/no)	1.353 (0.893–2.049)	0.154		
Surgery approach (left/right)	1.164 (0.810–1.672)	0.413		
Tumor location				
Upper	1.000			
Middle	0.826 (0.509–1.339)	0.437		
Lower	0.782 (0.427–1.432)	0.425		
TNM stage (I/II/III/IV)^a^				
I	1.000		1.000	
II	1.952 (0.998–3.819)	0.051	1.768 (0.891–3.510)	0.103
III	2.409 (1.255–4.624)	0.008*	2.133 (1.070–4.251)	0.031*
IV	3.288 (1.483–7.287)	0.003*	2.316 (1.010–5.310)	0.047*
Macroscopic types				
Ulcerative type	1.000		1.000	
Fungating type	1.459 (0.941–2.261)	0.091	1.781 (1.130–2.808)	0.013
Narrow type	1.255 (0.662–2.378)	0.486	1.229 (0.642–2.354)	0.533
Medullary type	1.975 (1.204–3.239)	0.007*	2.167 (1.298–3.620)	0.003*
Disease‐free duration (≤1 year/>1 year)	0.601 (0.418–0.864)	0.006*	0.692 (0.468–1.024)	0.066
Recurrence pattern (LNR/DM)	1.509 (1.043–2.183)	0.029*	1.682 (1.138–2.485)	0.009*
Salvage treatment (RT/CRT)	1.025 (0.702–1.499)	0.897		
Toxicity (Grade 1 and 2/Grade 3 and 4)	0.945 (0.656–1.360)	0.761		

Abbreviations: CI, confidence interval; CRT, chemoradiotherapy; DM, distant metastasis; ESCC, esophageal squamous cell cancer; HR, hazard ratio; LNR, lymph node recurrence; RT, radiotherapy.

^a^ TNM stage was evaluate by the 8th edition American Joint Committee on Cancer & The Union for International Cancer Control staging system.

* *p* < 0.050.

### 3.4 Subgroup analysis

**FIGURE 3:**
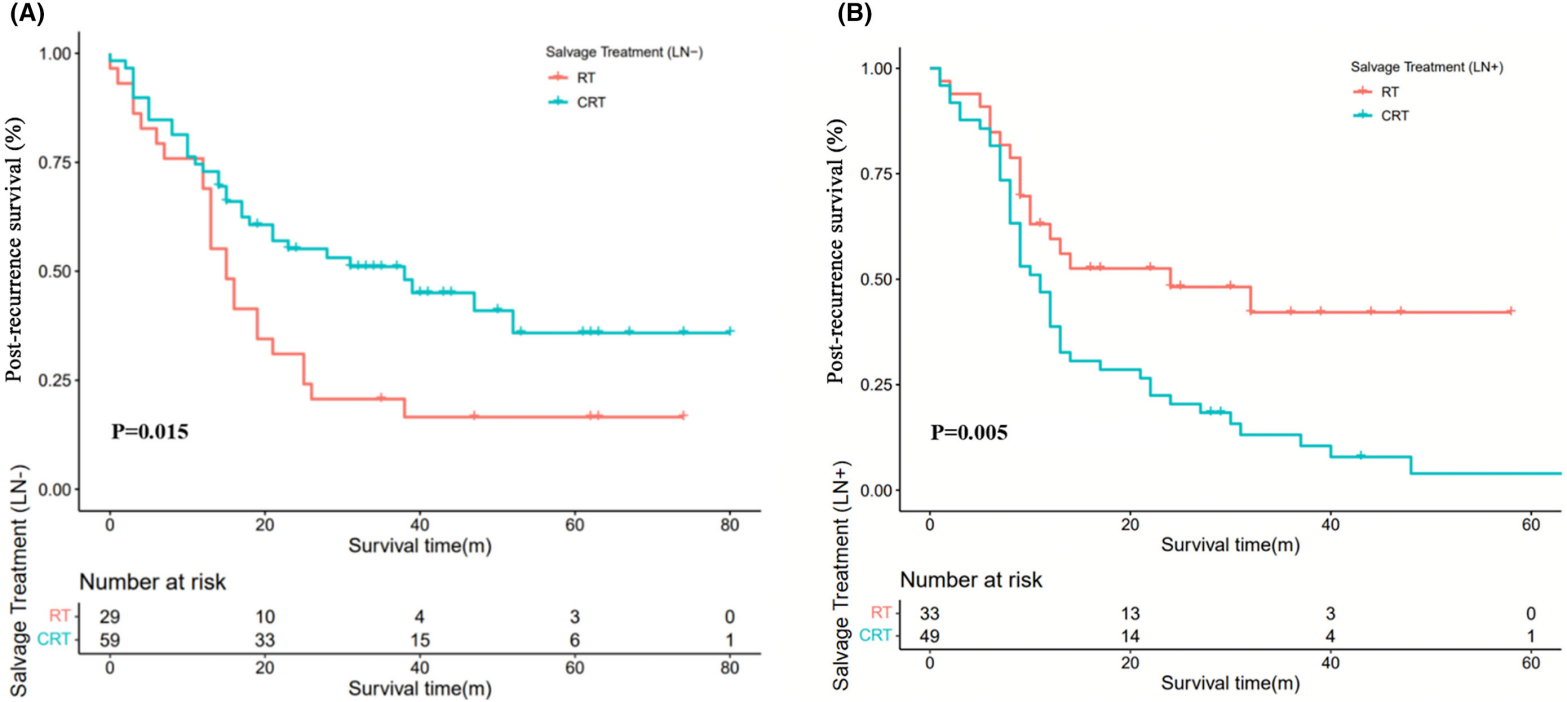
Kaplan–Meier curves of salvage treatment on PRS in negative LNs subgroup (A) and positive LNs subgroup (B). RT, radiotherapy; CRT, chemoradiotherapy; LN+, positive lymph node status; LN‐, negative lymph node status; PRS, post‐recurrence survival.

In the negative LN subgroup (88 patients), the PRS differed significantly between patients who received RT and CRT, with median survival times of 15 and 38 months, respectively (*p* = 0.015, Figure 3A). In the univariate analysis, salvage treatment and macroscopic type were related to survival after recurrence (*p* < 0.050). The multivariable analysis revealed that salvage CRT (HR = 0.557, 95% CI = 0.322–0.966, *p* = 0.037) was the only independent beneficial prognostic factor for PRS (Table 3).

**TABLE 3:** Cox regression analysis for ESCC patients with recurrence (LN−).

Parameters	Univariate		Multivariate	
	HR (95% CI)	*p*‐value	HR (95% CI)	*p‐*value
Sex (male/female)	0.846 (0.501–1.431)	0.533		
Age (≤60/>60)	1.000 (0.967–1.033)	0.977		
Neoadjuvant chemotherapy (yes/no)	1.040 (0.593–1.823)	0.890		
Adjuvant therapy (yes/no)	1.189 (0.700–2.019)	0.521		
Surgery approach (left/right)	1.321 (0.777–2.244)	0.304		
Tumor location				
Upper	1.000			
Middle	0.772 (0.394–1.510)	0.449		
Lower	0.403 (0.149–1.092)	0.074		
TNM stage^a^				
I	1.000			
II	1.908 (0.970–3.752)			
III	1.992 (0.691–5.748)			
IV	3.007 (0.831–10.876)			
Macroscopic types				
Ulcerative type	1.000		1.000	
Fungating type	1.534 (0.796–2.957)	0.201	1.335 (0.682–2.614)	0.399
Narrow type	1.687 (0.737–3.859)	0.216	1.442 (0.622–3.347)	0.394
Medullary type	2.160 (1.040–4.485)	0.039*	2.066 (0.990–4.311)	0.053
Disease‐free duration (≤1 year/>1 year)	0.730 (0.422–1.264)	0.262		
Recurrence pattern (LNR/DM)	1.020 (0.571–1.822)	0.948		
Salvage treatment (RT/CRT)	0.529 (0.311–0.900)	0.019*	0.557 (0.322–0.966)	0.037*
Toxicity (Grade 1 and 2/Grade 3 and 4)	0.696 (0.400–1.212)	0.200		

Abbreviations: CI, confidence interval; CRT, chemoradiotherapy; DM, distant metastasis; ESCC, esophageal squamous cell cancer; HR, hazard ratio; LN−, negative lymph node status; LNR, lymph node recurrence; RT, radiotherapy.

^a^ TNM stage was evaluate by the 8th edition American Joint Committee on Cancer & The Union for International Cancer Control staging system.

* *p* < 0.050.

However, for the positive LN subgroup (82 patients), patients who received RT showed a longer PRS than those who received CRT (24 months vs. 11 months, *p* = 0.005, Figure 3B). Univariate analysis indicated that the factors related to the PRS included salvage treatment and recurrence pattern (*p* < 0.050). Furthermore, distant metastasis (HR = 1.820, 95% CI = 1.094–3.028, *p* = 0.021) and salvage CRT (HR = 2.007, 95% CI = 1.142–3.526, *p* = 0.015) were found to be independent prognostic adverse factors for PRS in the multivariable analysis (Table 4).

**TABLE 4:** Cox regression analysis for ESCC patients with recurrence (LN+).

Parameters	Univariate		Multivariate	
	HR (95% CI)	*p*‐value	HR (95% CI)	*p*‐value
Sex (male/female)	0.654 (0.391–1.094)	0.106		
Age (≤60/>60)	1.013 (0.980–1.048)	0.432		
Neoadjuvant chemotherapy (yes/no)	1.524 (0.848–2.740)	0.159		
Adjuvant therapy (yes/no)	0.709 (0.280–1.797)	0.469		
Surgery approach (left/right)	1.002 (0.606–1.658)	0.993		
Tumor location				
Upper	1.000			
Middle	0.857 (0.425–1.728)	0.667		
Lower	1.220 (0.545–2.731)	0.628		
TNM stage^a^				
II	1.000			
III	0.239 (0.057–1.004)	0.051		
IV	0.327 (0.071–1.502)	0.151		
Macroscopic types				
Ulcerative type	1.000			
Fungating type	1.369 (0.759–2.471)	0.297		
Narrow type	1.026 (0.356–2.958)	0.963		
Medullary type	1.888 (0.960–3.713)	0.066		
Disease‐free duration (≤1 year/>1 year)	0.597 (0.350–1.018)	0.058		
Recurrence pattern (LNR/DM)	1.975 (1.191–3.275)	0.008*	1.820 (1.094–3.028)	0.021*
Salvage treatment (RT/CRT)	2.158 (1.232–3.777)	0.007*	2.007 (1.142–3.526)	0.015*
Toxicity (Grade 1 and 2/Grade 3 and 4)	1.200 (0.723–1.993)	0.481		

Abbreviations: CI, confidence interval; CRT, chemoradiotherapy; DM, distant metastasis; ESCC: esophageal squamous cell cancer; HR: hazard ratio; LN+, positive lymph node status; LNR, lymph node recurrence; RT, radiotherapy.

^a^ TNM stage was evaluate by the 8th edition American Joint Committee on Cancer & The Union for International Cancer Control staging system.

* *p* < 0.050.

**FIGURE 4:**
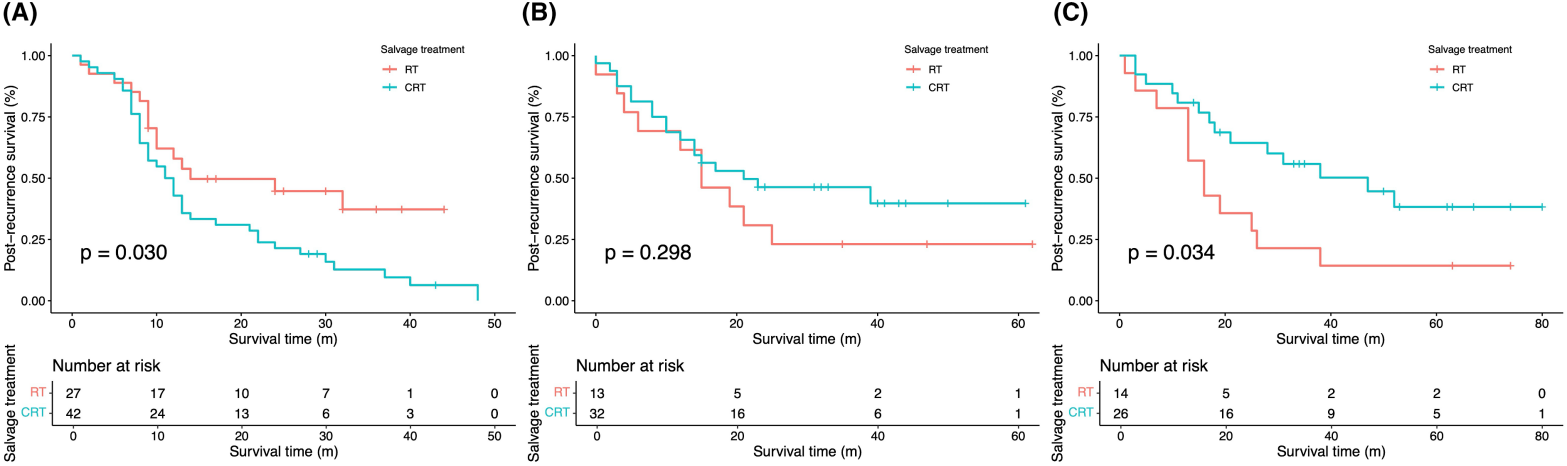
Kaplan–Meier curves of salvage treatment on PRS in positive LNs subgroup with adjuvant chemotherapy, (A) negative LNs subgroup with adjuvant chemotherapy (B) and without adjuvant chemotherapy (C). RT, radiotherapy; CRT, chemoradiotherapy; LNs, Lymph nodes; PRS, post‐recurrence survival.

LN‐negative patients without adjuvant treatment showed better survival in the salvage CRT group (*p* = 0.034); for LN‐positive patients with adjuvant CT, the salvage RT group demonstrated better PRS (*p* = 0.030, Figure 4). The survival curves for the RT and CRT groups showed no significant difference in patients with LN recurrence and in patients with distant metastases (*p* = 0.820 and *p* = 0.910, respectively, Figure S3). In the salvage CRT group, the survival rate for single‐site LN recurrence was significantly better than for multi‐site distant metastasis (*p* = 0.015, Figure S4). The survival curves for patients who received and did not receive neoadjuvant CT in the salvage RT and CRT groups showed no significant differences (*p* = 0.055 and *p* = 0.640, respectively, Figure S5).

### 3.5 Risk factors for toxicity reactions

Among all patients, only salvage treatment may be associated with toxicity reaction (HR = 1.760, 95%CI = 0.929–3.333, *p* = 0.083, Table S1). For the toxicity analysis based on LN status subgroups, CRT (HR = 2.867, 95% CI = 1.106–7.436, *p* = 0.030) and male sex (HR = 0.350, 95% CI = 0.136–0.899, *p* = 0.029) were found to be independent risk factors for grade 3/4 toxicity in the positive LN groups. Nevertheless, there was no significant difference between salvage treatment methods regarding toxic reactions in the negative LN subgroup (Tables S2 and S3).

## 4 DISCUSSION

Considering the controversial clinical effect of salvage RT and CRT, we attempted to specify the optimal regimen for patients with recurrent ESCC and further performed subgroup analyses based on postoperative LN status to identify differences in efficacy and toxicity between RT and CRT. The following findings were revealed: (a) advanced TNM stage III and IV, macroscopic medullary type, and distant metastasis were independently associated with poor PRS; (b) CRT was associated with prolonged survival for patients with postoperative negative LNs, while RT showed better clinical benefits for patients with positive LNs; and (c) in terms of toxicity, RT had lower toxic reactions than CRT in the positive LN group, whereas there were no significant differences in the negative LN group.

In the current study, the left thoracic approach was undergone for esophagectomy in 46.5% patients, and this may have contributed to the higher number of LN recurrences due to inadequate removal of the superior mediastinal LNs. Advanced TNM stage was associated with poor survival after recurrence both in our and previous studies.^2^, ^23^ DFS (≤1/>1 year) has been reported as an independent prognostic factor^23^, ^24^, ^25^; however, it was not found to be significant in current study. The shorter the DFS was, the worse the patient's prognosis after relapse, and patients who relapsed within 1 year of surgery had a median survival duration of 5.9–15 months.^25^, ^26^, ^27^, ^28^ A DFS of more than 1 year may suggest a relatively successful esophagectomy and a possibility for a manageable intervention even after recurrence. Moreover, distant metastasis is recognized as another factor for poor prognosis after recurrence.^12^, ^29^ Patients with multiple distant metastases had a significantly worse median survival than those with LN recurrence.^12^ In addition, tumors that develop hematogenous distant recurrence would reflect more aggressive tumor biology, with higher metastatic potential than locoregional recurrence.^26^ Interestingly, in our study, the patients with the medullary macroscopic type showed poor survival, which has not been reported previously. Tumor macroscopic type may play an important role in survival, as reflected by LN metastasis and sensitivity to CRT.^30^

Although CRT performed better than RT due to radiation sensitization and antitumor factors,^2^, ^31^, ^32^ in the whole cohort of our study, there was no significant difference in clinical outcomes between patients receiving salvage RT and those receiving salvage CRT. Yamashita et al.^8^ observed that the 3‐year PRS rate was substantially higher in the CRT group than in the RT group (39.7% vs. 20.8%) among patients with recurrent ESCC. Other studies have reported similar findings that CRT led to a better prognosis than RT alone.^2^, ^31^, ^32^ The reason for these differences may be that the previous studies included only patients with LN recurrence, whereas we also included patients with distant metastases, which have been shown to indicate a poor effect.^12^, ^27^ However, our results showed that no difference in PRS between CRT and RT in patients with LN recurrence (Figure S3A). Chen et al.^18^ even reported higher 3‐year PRS rates of 47.5% and 41.9% in patients receiving RT and CRT, respectively. The better prognosis of the patients in that study are attributed to intensity‐modulated radiation therapy and volumetric modulated arc therapy, which have the characteristics of adjustable radiation intensity and precise localization of the recurrence area.

It is worth noting that patients with diverse postoperative LN statuses who received different salvage therapies after relapse had significantly different survival outcomes in our subgroups. CRT was associated with prolonged survival in the subgroup of patients with negative LNs. In contrast, RT showed better clinical benefits than CRT for patients with positive LNs. We speculated that the current results were related to the differences in postoperative adjuvant treatments received by patients with different LN statuses. In LN‐positive patients, the majority (84%) received adjuvant CT. Upon recurrence, those who underwent salvage CRT experienced increased resistance and decreased sensitivity, along with heightened toxicity,^33^ which were associated with poorer prognosis (Figure 4A). These issues were not a concern in patients with pathological LN‐negative status who did not receive adjuvant CT (45.5%). Consequently, they benefited more from salvage CRT after recurrence, achieving better PRS (Figure 4B). Pathological LN status, which influences the choice of both postoperative adjuvant therapy and recurrence treatment, necessitates accurate pathological assessment.^20^, ^34^

Although effective radiation control and concurrent CT could lead to amplified damage to tumor cells and inhibit tumor cell proliferation kinetics, more attention needs to be given to chemotherapeutic toxicity and radiation injury.^35^ To eradicate recurrent tumors more aggressively, we performed RT at doses 50–60 Gy, with a grade 3/4 toxicity rate of 45.9%. The tolerated dose of adjacent normal tissue, such as the heart, lungs, liver, and spinal cord, continues to be the major issue of further salvage treatment. A higher grade of toxicity may be related to a heavy tumor burden, a large target area, and poor status of patients with recurrent ESCC undergoing reirradiation. Furthermore, the grade 3/4 toxicity effects of severe vomiting and esophageal damage occurred more frequently in the CRT group due to the application of chemotherapeutic drugs.^35^ Precise and reliable dose distributions that allows for appropriate proper radiation therapy to the tumor while limiting radiation exposure to surrounding normal tissues can be effective in reducing these side effects.^36^ Nevertheless, among all patients, no significant difference in toxic reactions between salvage RT and CRT was found in our study. However, patients with positive LN receiving CRT had a considerably higher toxic reactions, but no difference in toxicity was observed in the negative LN group. Haque et al.^37^ mentioned that patients with positive LNs at the time of primary surgical resection were likely to have relatively unfavorable outcomes, which may lead to poor esophageal function and result in a higher rate of toxic reactions. Gao et al.^38^ mentioned that RT alone may offer lower toxicity, better survival, and better quality of life and might be the recommended choice of treatment for patients with positive LNs. Therefore, for recurrence ESCC patients with positive LNs, poor health, and complications, RT should be recommended to avoid the occurrence of severe toxic reactions and provide better survival and quality of life.

The limitations of the current study are as follows. First, inevitable selection bias existed in this retrospective study. A randomized controlled trial should further validate the current results. Second, the type of postoperative adjuvant therapy and the physical condition of the patients may lead to discrepancies in the clinical effect of salvage treatments, and a larger prospective, multicenter study is required to confirm our findings. Additionally, immunotherapy after relapse may be valuable as an expanded treatment modality, and future prospective studies on this topic are expected.

## 5 CONCLUSION

To achieve a better prognosis and fewer toxic reactions, ESCC patients with postoperative recurrence and negative LNs may receive CRT, while RT should be recommended for patients with positive LNs. Our results may help determine the optimal salvage treatment strategy for individuals in the clinic.

## AUTHOR CONTRIBUTIONS

**Qing Liu:** Conceptualization (equal); methodology (equal); writing – original draft (equal). **Xue‐Hua Tu:** Conceptualization (equal); methodology (equal); writing – original draft (equal). **Rui‐Xuan Yu:** Data curation (equal); formal analysis (equal). **Hong‐Ying Wen:** Data curation (equal); formal analysis (equal). **Xiao‐Guang Guo:** Data curation (equal); formal analysis (equal). **Dai‐Yuan Ma:** Supervision (equal); writing – review and editing (equal). **Kai‐Yuan Jiang:** Supervision (equal); writing – review and editing (equal). **Dong Tian:** Supervision (equal); writing – review and editing (equal).

## FUNDING INFORMATION

This study was funded by 2023 Clinical Research Fund, West China Hospital, Sichuan University (Dong Tian, No. 2023HXFH042); Youth Innovation Scientific Research Project of Sichuan Medical Association (Hong‐Ying Wen, No. Q21040); Preliminary Research Program of National Natural Science Foundation, North Sichuan Medical College (Hong‐Ying Wen, No. 20SXZRKX0001).

## CONFLICT OF INTEREST STATEMENT

The authors declare no conflict of interest.

## Supporting information


**Data S1:** Supporting Information.

## Data Availability

Data supporting the results of this research can be obtained from the corresponding author for specific request.
