# Supplementary material for: Radiotherapy and chemoradiotherapy for postoperative recurrence in patients with esophageal squamous cell carcinoma
Source: Cancer Med. 2024 Aug 19;13(16):e70108. doi: 10.1002/cam4.70108 (PMC11333533; doi:10.1002/cam4.70108)
Supplement: Supplementary file 1 — Data S1: Supporting Information. [file CAM4-13-e70108-s001.docx]

**Supplementary Table 1:** **Logistic Regression of Toxicity for ESCC patients with recurrence**

| **Parameters** | **Univariate analysis** | | | **Multivariate analysis** | | |
| --- | --- | --- | --- | --- | --- | --- |
|  | **HR** | **95% CI** | ***P*** | **HR** | **95% CI** | ***P*** |
| Sex (male/female) | 0.696 | 0.378-1.279 | 0.243 |  |  |  |
| Age (continue) | 0.986 | 0.948-1.025 | 0.475 |  |  |  |
| Neoadjuvant chemotherapy (yes/no) | 1.044 | 0.527-2.067 | 0.902 |  |  |  |
| [Adjuvant therapy (yes/no)](#keyfrom=E2Ctranslation) | 1.142 | 0.578-2.258 | 0.702 |  |  |  |
| Surgery approach (left/right) | 1.239 | 0.676-2.272 | 0.488 |  |  |  |
| Tumor location |  |  |  |  |  |  |
| upper | 1.000 |  |  |  |  |  |
| middle | 1.071 | 0.463-2.475 | 0.872 |  |  |  |
| lower | 1.511 | 0.544-4.194 | 0.428 |  |  |  |
| TNM stage ^a^ |  |  |  |  |  |  |
| I | 1.000 |  |  |  |  |  |
| II | 0.657 | 0.249-1.734 | 0.396 |  |  |  |
| III | 1.390 | 0.552-3.503 | 0.484 |  |  |  |
| IV | 0.919 | 0.257-3.283 | 0.897 |  |  |  |
| Macroscopic types ^b^ |  |  |  |  |  |  |
| ulcerative type | 1.000 |  |  |  |  |  |
| fungating type | 1.222 | 0.587-2.546 | 0.592 |  |  |  |
| constrictive type | 1.037 | 0.350-3.072 | 0.948 |  |  |  |
| medullary type | 1.422 | 0.616-3.283 | 0.409 |  |  |  |
| Disease-free survival  (≤1 year/＞1 year) | 0.664 | 0.362-1.219 | 0.186 |  |  |  |
| Recurrence pattern (LNR/DM) | 1.358 | 0.720-2.559 | 0.344 |  |  |  |
| Salvage treatment (RT/CRT) | 1.760 | 0.929-3.333 | 0.083 |  |  |  |

* P<0.050; a: TNM stage was evaluate by the 8th edition American Joint Committee on Cancer & The Union for International Cancer Control staging system; RT, radiotherapy; CRT, chemoradiotherapy.

**Supplementary Table 2:** **Logistic regression of toxicity for ESCC patients with recurrence (LN-)**

| **Parameters** | **Univariate analysis** | | | | **Multivariate analysis** | | |
| --- | --- | --- | --- | --- | --- | --- | --- |
|  | **HR** | **95% CI** | | ***P*** | **HR** | **95% CI** | ***P*** |
| Sex (male/female) | 1.583 | | 0.667-3.756 | 0.297 |  |  |  |
| Age (continue) | 0.979 | | 0.928-1.033 | 0.443 |  |  |  |
| Neoadjuvant chemotherapy (yes/no) | 1.136 | | 0.450-2.867 | 0.787 |  |  |  |
| [Adjuvant therapy (yes/no)](#keyfrom=E2Ctranslation) | 0.611 | | 0.257-1.451 | 0.264 |  |  |  |
| Surgery approach (left/right) | 1.974 | | 0.816-4.774 | 0.131 |  |  |  |
| Tumor location (upper/middle/lower) |  | |  |  |  |  |  |
| upper | 1.000 | |  |  |  |  |  |
| middle | 1.158 | | 0.353-3.797 | 0.809 |  |  |  |
| lower | 3.960 | | 0.865-18.119 | 0.076 |  |  |  |
| TNM stage (I/II/III/IV) ^a^ |  | |  |  |  |  |  |
| I | 1.000 | |  |  |  |  |  |
| II | 0.642 | | 0.241-1.706 | 0.374 |  |  |  |
| III | 0.886 | | 0.162-4.846 | 0.889 |  |  |  |
| IV | 0.591 | | 0.047-7.427 | 0.684 |  |  |  |
| Macroscopic types ^b^ |  | |  |  |  |  |  |
| ulcerative type | 1.000 | |  |  |  |  |  |
| fungating type | 1.236 | | 0.423-3.613 | 0.698 |  |  |  |
| constrictive type | 1.448 | | 0.384-5.770 | 0.565 |  |  |  |
| medullary type | 1.071 | | 0.321-3.576 | 0.911 |  |  |  |
| Disease-free survival  (≤1 year/＞1 year) | 0.487 | | 0.198-1.200 | 0.118 |  |  |  |
| Recurrence pattern (LNR/DM) | 1.314 | | 0.500-3.453 | 0.579 |  |  |  |
| Salvage treatment (RT/CRT) | 1.045 | | 0.419-2.609 | 0.924 |  |  |  |

* P<0.050; a: TNM stage was evaluate by the 8th edition American Joint Committee on Cancer & The Union for International Cancer Control staging system; LN-, negative lymph node status; RT, radiotherapy; CRT, chemoradiotherapy.

**Supplementary Table 3:** **Logistic regression of toxicity for ESCC patients with recurrence (LN+)**

| **Parameters** | **Univariate analysis** | | | | **Multivariate analysis** | | |
| --- | --- | --- | --- | --- | --- | --- | --- |
|  | **HR** | **95% CI** | | ***P*** | **HR** | **95% CI** | ***P*** |
| Sex (male/female) | 0.305 | 0.122-0.760 | 0.011* | | 0.350 | 0.136-0.899 | 0.029* |
| Age (continue) | 0.989 | 0.932-1.050 | 0.728 | |  |  |  |
| Neoadjuvant chemotherapy (yes/no) | 1.103 | 0.385-3.156 | 0.855 | |  |  |  |
| [Adjuvant therapy (yes/no)](#keyfrom=E2Ctranslation) | 2.471 | 0.427-14.311 | 0.313 | |  |  |  |
| Surgery approach (left/right) | 0.810 | 0.339-1.934 | 0.634 | |  |  |  |
| Tumor location (upper/middle/lower) |  |  |  | |  |  |  |
| upper | 1.000 |  |  | |  |  |  |
| middle | 0.901 | 0.253-3.212 | 0.872 | |  |  |  |
| lower | 0.571 | 0.130-2.503 | 0.458 | |  |  |  |
| TNM stage (I/II/III/IV) ^a^ |  |  |  | |  |  |  |
| II | 1.000 |  |  | |  |  |  |
| III | 1.233 | 0.074-20.554 | 0.884 | |  |  |  |
| IV | 0.857 | 0.044-16.851 | 0.919 | |  |  |  |
| Macroscopic types ^b^ |  |  |  | |  |  |  |
| ulcerative type | 1.000 |  |  | |  |  |  |
| fungating type | 1.182 | 0.424-3.291 | 0.749 | |  |  |  |
| constrictive type | 0.667 | 0.100-4.452 | 0.676 | |  |  |  |
| medullary type | 2.000 | 0.574-6.965 | 0.276 | |  |  |  |
| Disease-free survival  (≤1 year/＞1 year) | 1.187 | 0.486-2.896 | 0.707 | |  |  |  |
| Recurrence pattern (LNR/DM) | 1.146 | 0.477-2.751 | 0.761 | |  |  |  |
| Salvage treatment (RT/CRT) | 3.294 | 1.311-8.278 | 0.011* | | 2.867 | 1.106-7.436 | 0.030* |

* P<0.050; a: TNM stage was evaluate by the 8th edition American Joint Committee on Cancer & The Union for International Cancer Control staging system; LN+, positive lymph node status; RT, radiotherapy; CRT, chemoradiotherapy.

**Supplementary Figure 1:** **Kaplan-Meier curves of LNR at single and multiple sites (A) and DM at single and multiple sites (B).**

**
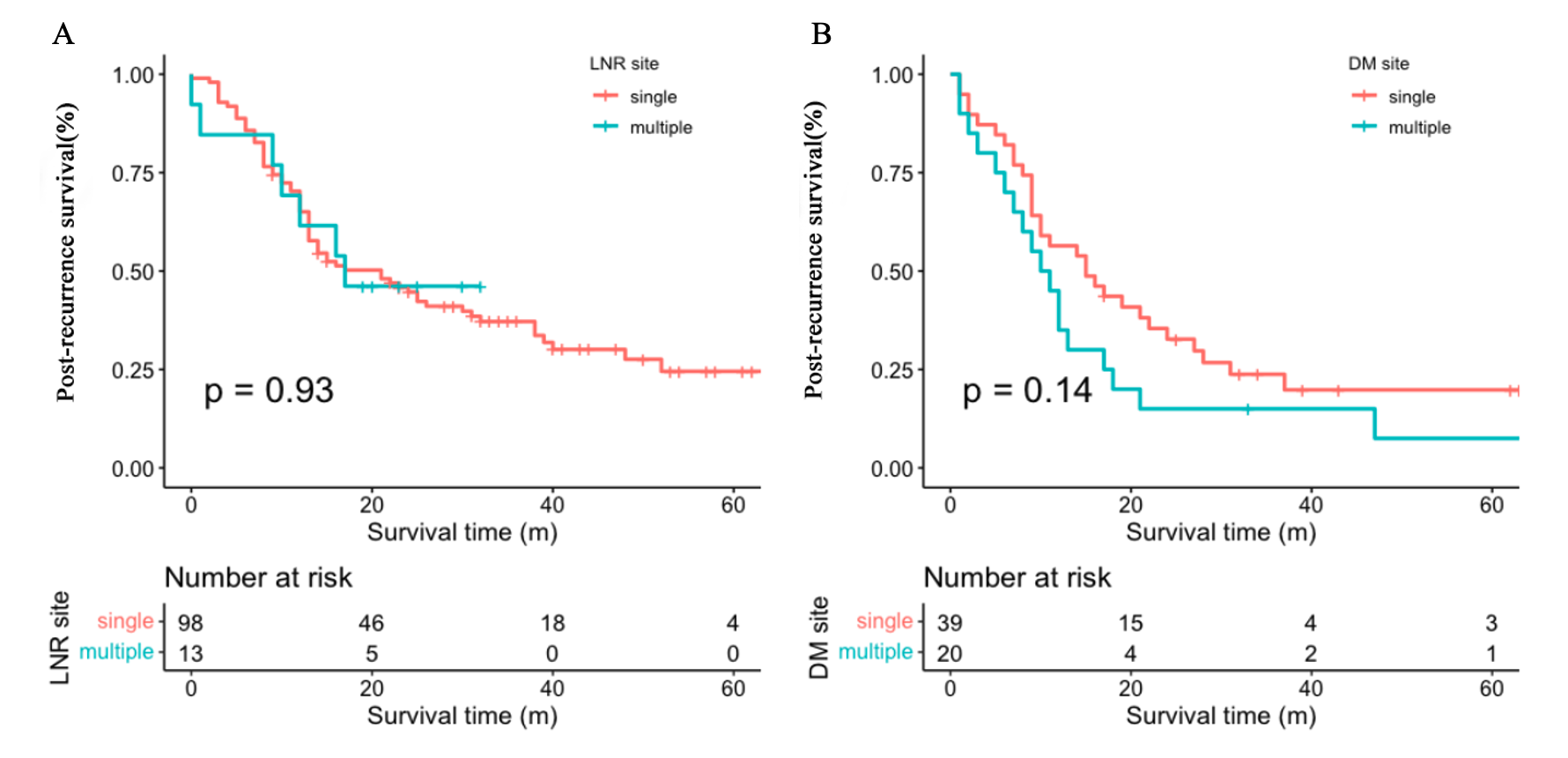
**

LNR, lymph node recurrence; DM, distant metastasis.

**Supplementary Figure 2: Kaplan-Meier curves of TNM (A), macroscopic types (B), DFS (C) and recurrence pattern (D)**


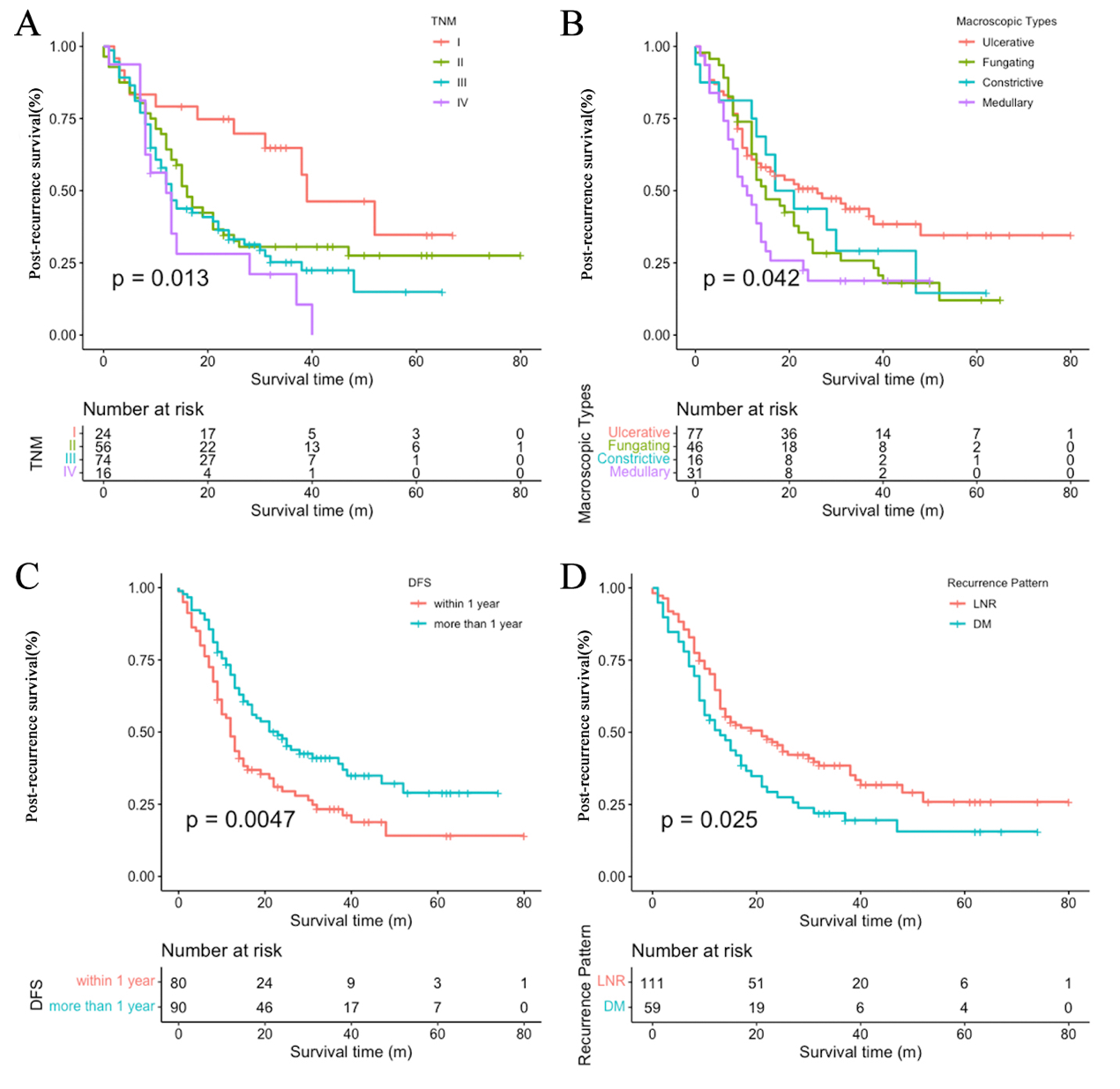


TNM, TNM stage was evaluate by the 8th edition American Joint Committee on Cancer & The Union for International Cancer Control staging system; DFS, disease-free survival; LNR, lymph node recurrence; DM, distant metastasis.

**Supplementary Figure 3: Kaplan-Meier curves of salvage treatment in patients with LN recurrence (A) and distant metastasis (B).**


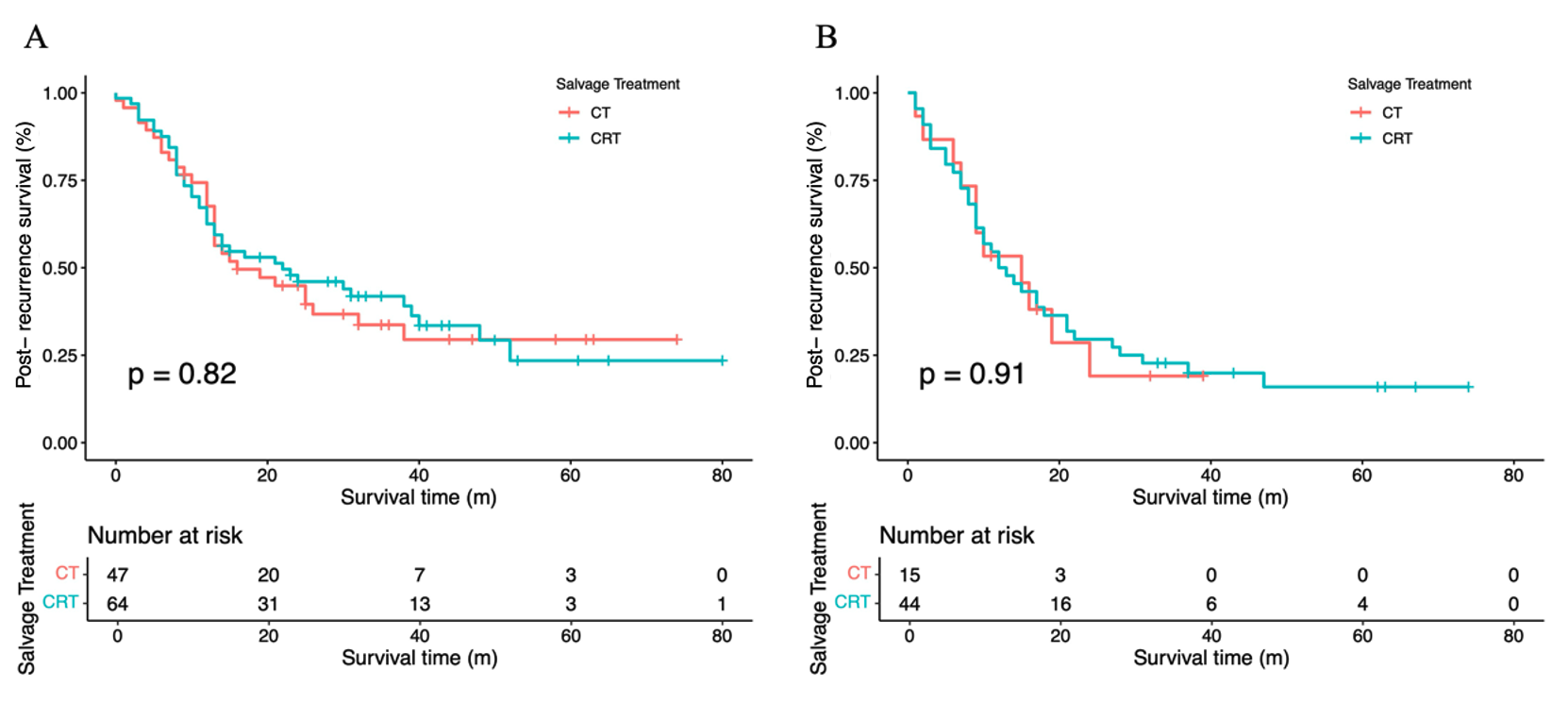


LN, lymph node; RT, radiotherapy; CRT, chemoradiotherapy.

**Supplementary Figure 4: Kaplan-Meier curves of recurrence patterns for patients receiving salvage RT (A) and CRT (B).**


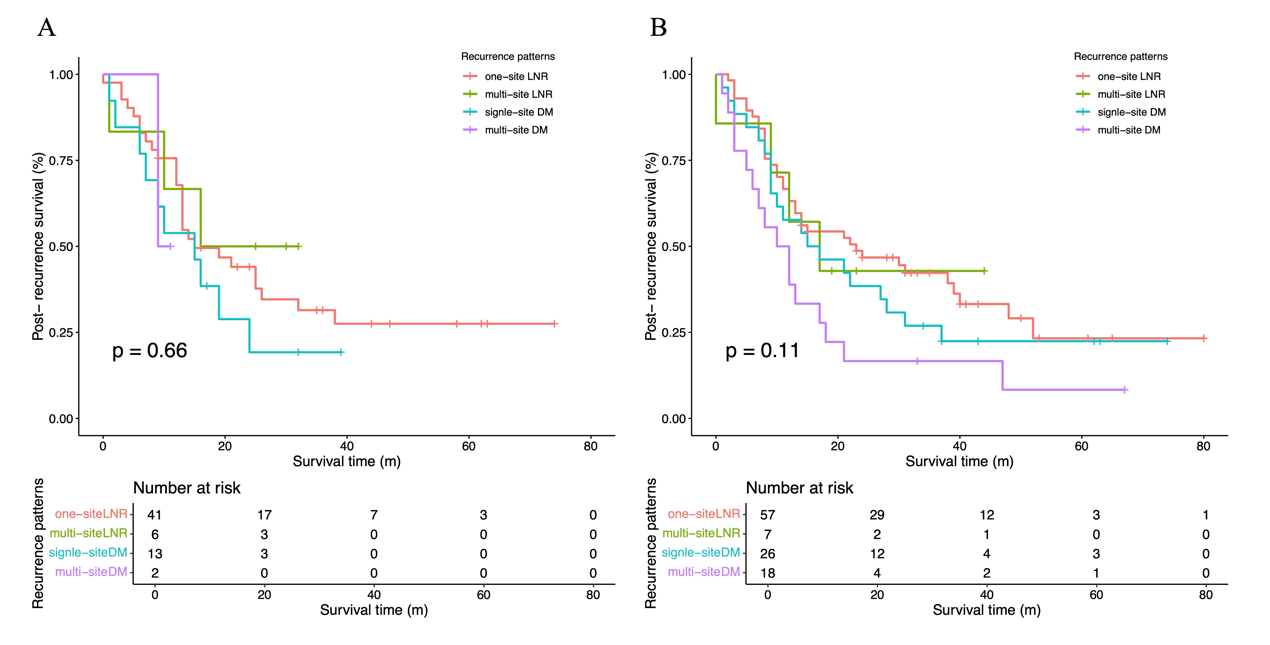


LNR, lymph node recurrence; DM, distant metastasis; RT, radiotherapy; CRT, chemoradiotherapy. (A): one-site LNR vs. multi-site LNR: P=0.615; one-site LNR vs. one-site DM: P=0.322; one-site LNR vs. multi-site DM: P=0.566; multi-site LNR vs. one-site DM: P=0.291; multi-site LNR vs. multi -site DM: P=0.683; one-site DM vs. multi -site DM: P=0.939. (B): one-site LNR vs. multi-site LNR: P=0.910; one-site LNR vs. one-site DM: P=0.355; one-site LNR vs. multi-site DM: P=0.015; multi-site LNR vs. one-site DM: P=0.706; multi-site LNR vs. multi -site DM: P=0.252; one-site DM vs. multi -site DM: P=0.165.

**Supplementary Figure 5: Kaplan-Meier curves** **of patients who received and did not receive neoadjuvant chemotherapy in the salvage RT (A) and CRT (B) groups.**


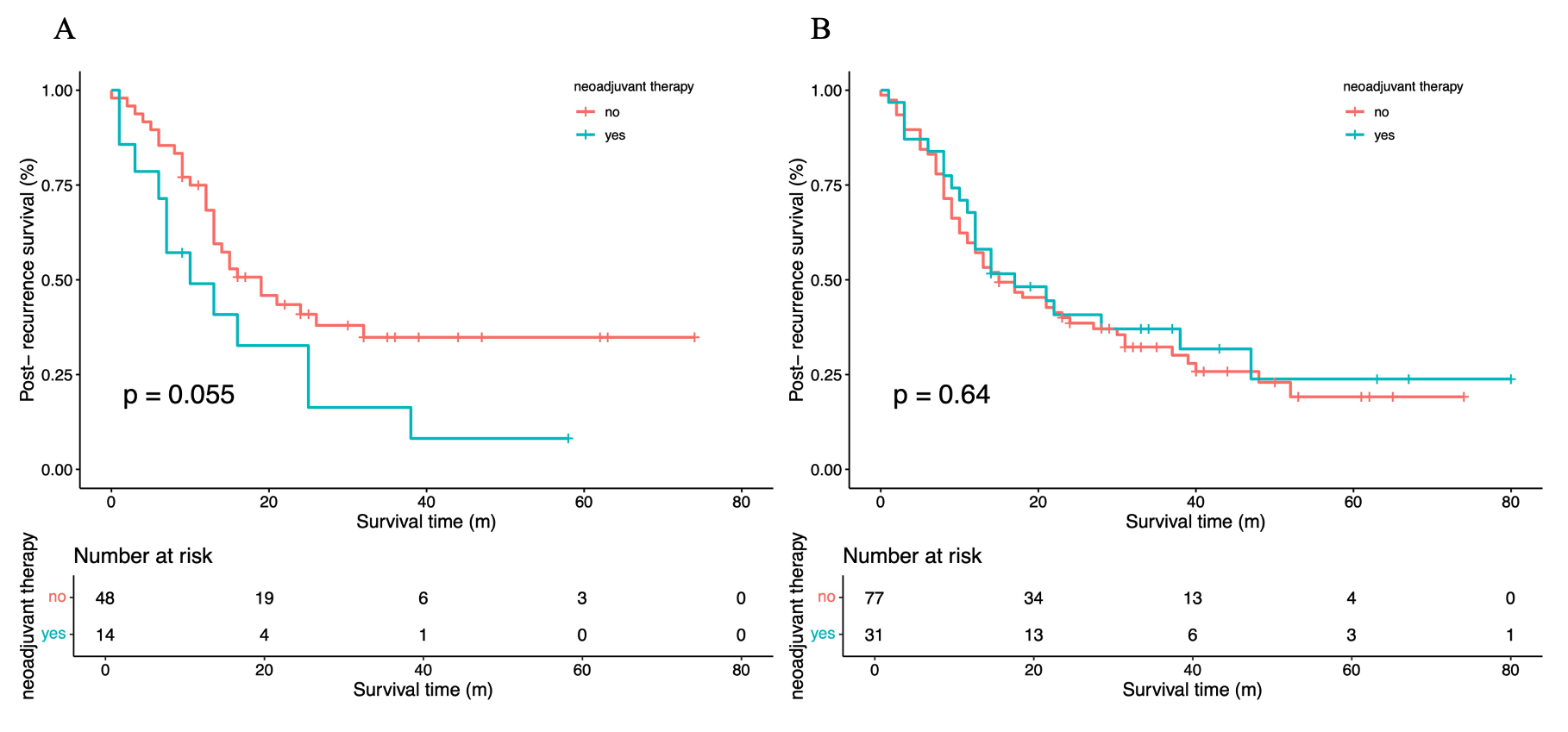


RT, radiotherapy; CRT, chemoradiotherapy.
